# Assessing the impact of binge drinking and a prebiotic intervention on the gut–brain axis in young adults: protocol for a randomised controlled trial

**DOI:** 10.1136/bmjopen-2024-095932

**Published:** 2025-09-04

**Authors:** Diogo Prata-Martins, Clarisse Nobre, Natália Almeida-Antunes, Pedro Azevedo, Sónia S Sousa, Alberto Crego, John Cryan, Adriana Sampaio, Carina Carbia, Eduardo López-Caneda

**Affiliations:** 1 School of Psychology, University of Minho, Braga, Portugal; 2 University of Minho, Braga, Portugal; 3 LABBELS - Associate Laboratory, Braga Guimarães, Portugal; 4 Health Research Network (RISE-Health)/Center for Translational Health and Medical Biotechnology Research (TBIO), ESS, Polytechnic of Porto, R. Dr. António Bernardino de Almeida, 400, 4200 - 072, Porto, Portugal; 5 Psychologial Neuroscience Laboratoy (PNL), Psychology Reseach Center (CIPSI), School of Psychology, University of Minho, Braga, Portugal; 6 Psychology Research Center, Braga, Portugal; 7 Anatomy & Neuroscience, University College Cork, Cork, Ireland; 8 Louvain Experimental Psychopathology Research Group, Brussels, Belgium; 9 Psychologial Neuroscience Laboratoy (PNL), Psychology Research Center (CIPSI), School of Psychology, University of Minho, Braga, Portugal

**Keywords:** Adolescents, Substance misuse, Clinical Trial, Microbiota, Magnetic Resonance Imaging

## Abstract

**Introduction:**

Adolescence and youth are periods of significant maturational changes, which seem to involve greater susceptibility to disruptive events in the brain, such as binge drinking (BD). This pattern—characterised by repeated episodes of alcohol intoxication—is of particular concern, as it has been associated with significant alterations in the developing brain. Recent evidence indicates that alcohol may also induce changes in gut microbiota composition and that such disturbances can lead to impairments in both brain function and behaviour. Moreover, there is evidence suggesting that microbiota-targeted interventions (psychobiotics) may help mitigate alcohol-induced damage in individuals with chronic alcohol use, positively influencing cognitive and brain functioning. However, the triadic relationship between BD, gut microbiota and brain structure/function, as well as the therapeutic potential of gut microbiota-targeted interventions in young binge drinkers, remains largely unexplored.

**Methods and analysis:**

This double-blind, parallel, randomised controlled study aims to evaluate whether a BD pattern disrupts gut microbiota diversity in young college students (primary outcome). Additionally, it seeks to determine whether alcohol-induced alterations in the microbial composition and function are associated with immunological, cognitive, neurostructural and neurofunctional impairments (secondary outcomes). A total of 82 college students (36 non/low drinkers and 46 binge drinkers (BDs)), matched for age and sex, will be recruited from the University of Minho (Portugal). During the pre-intervention phase, all participants will undergo a comprehensive assessment protocol, including gut microbiota profiling, measurement of inflammatory markers, neuropsychological testing and structural and functional MRI. BDs will then be randomly assigned to a 6-week intervention with either a prebiotic (inulin) or a placebo (maltodextrin). Post-intervention assessment will mirror the baseline protocol, and craving and alcohol use will be monitored for 3 months.

**Ethics and dissemination:**

The present protocol was approved by the Ethics Committee for Social and Human Sciences of the University of Minho (CEICSH 078/2022), ensuring compliance with national and international ethical guidelines, including the Declaration of Helsinki. Participation is voluntary and preceded by informed consent, with confidentiality and data processing safeguarded in accordance with the General Data Protection Regulation. All procedures are safe and non-invasive, and the prebiotics used are recognised as food ingredients in Europe, hold Generally Recognized as Safe status in the USA and are classified as dietary fibres by the Food and Drug Administration. Findings will be disseminated in national and international scientific forums, with preference for publication in open-access, peer-reviewed journals.

**Trial registration number:**

NCT05946083

**STRENGTHS AND LIMITATIONS OF THIS STUDY**

- Randomised, double-blind, placebo-controlled design applied in a vulnerable young adult population with binge alcohol use.
- Multidisciplinary protocol including gut microbiota sequencing, neuroimaging, and immune and cognitive assessments.
- Age-matched and sex-matched control and intervention groups enhance comparability.
- Potential low adherence to the 6-week intervention among binge drinkers may affect data quality.
- Lifestyle factors such as exercise, sleep and stress are not fully controlled throughout the study.

## Introduction

 A prevalent form of alcohol misuse among young people that has garnered considerable attention in the last two decades is binge drinking (BD), characterised by episodes of excessive alcohol consumption followed by periods of low intake or abstinence.^1 2^ According to the National Institute on Alcohol Abuse and Alcoholism, BD is defined as a pattern of alcohol use that results in a blood alcohol concentration level of 0.08 g/dL, typically occurring after consuming a minimum of four (for women) or five (for men) alcoholic beverages within a span of 2 hours, at least once a month.^3^

Concern over the rising prevalence of BD among young people has emerged as a critical public health issue, particularly due to the heightened vulnerability of this age group to the neurotoxic effects of alcohol.^4^ In most Western countries, including Portugal, this high consumption pattern is a regular practice in approximately one-third of youths aged 15–24 years.^5^ The implications of this behaviour extend beyond immediate physical and quality-of-life issues, encompassing neurocognitive consequences.^6^,^8^ Research focusing on the long-term impacts on young BDs has identified alterations in both brain structure and function, highlighting the severity and complexity of the risks associated with excessive alcohol use in this stage of development.^9 10^

Accumulating evidence indicates that alcohol misuse induces inflammation both directly in the brain, for instance, via pro-inflammatory cytokines, and indirectly through systemic mechanisms, such as those associated with gut microbiota alterations.^11 12^ This complex microbial ecosystem comprises over 100 trillion microorganisms primarily residing in the lower intestine, existing in a symbiotic relationship with the host.^13^ It plays a crucial role in nutrient metabolism—fermenting non-digestible substrates such as prebiotics and dietary fibre to produce microbial metabolites like short-chain fatty acids (SCFAs)—while also regulating immune function and contributing to the production of neurotransmitters, including serotonin, gamma-aminobutyric acid and dopamine, all of which are essential for maintaining overall homeostasis.^14^,^16^

Recent research has underscored the pivotal role of gut microbiota in mental health conditions, including alcohol use disorders.^12 17^ Accordingly, chronic alcohol use has been associated with impaired gut barrier function and increased intestinal permeability (‘leaky gut’). This disruption allows bacteria, toxins and microbial products such as lipopolysaccharides to translocate into the bloodstream, triggering inflammatory responses in both the gut and liver, which may, in turn, contribute to brain inflammation.^18 19^ In addition to the production of circulating inflammatory cytokines, alcohol consumption has been linked to metabolic alterations, including reduced levels of IgA and SCFAs, both of which are crucial for the regulation of microglial activity in the central nervous system.^19^ Altogether, these cumulative effects highlight the potential for alcohol abuse to disrupt the delicate balance between the gut microbiota, immune responses and brain health.

While inconsistencies remain, research on alcohol-related microbiome alterations suggests the presence of specific microbial taxonomic signatures linked to dysfunctions associated with excessive alcohol use. Preclinical studies have shown decreased gut microbial α-diversity and β-diversity—that is, diversity within and between microbial communities, respectively—in alcohol-exposed animals.^20^ In addition, several studies in animal models have reported differential relative abundance at the phylum level, including reductions in *Bacteroidetes* and increases in *Firmicutes*, resulting in an elevated *Firmicutes/Bacteroidetes* ratio.^21 22^ On the other hand, research involving individuals with alcohol use disorder (AUD) has similarly found reductions in α-diversity, as well as a depletion of beneficial bacteria from the *Bacteroidetes* and *Firmicutes* phyla (including the *Ruminococcaceae* and *Lachnospiraceae* families), together with a rise in pro-inflammatory bacterial groups, such as *Proteobacteria*.^23^,^26^ Although data remain limited, preclinical research on binge drinking has also indicated alcohol-induced microbial dysbiosis in rats, which persisted into adulthood.^27^ Similarly, a recent study involving young BDs reported gut microbiota alterations, which were in turn associated with alcohol craving and emotional and cognitive difficulties.^28^

Altogether, considering the relationship between AUD and disruptions in gut microbial composition, as well as recent finding showing gut microbiota changes in youths with heavy alcohol use,^28 29^ interventions targeting the gut microbiota may constitute a promising therapeutic approach, not only to minimise alcohol-induced damage^14 30^ but also to modify alcohol use patterns.^31^,^33^ In this context, prebiotics, probiotics, synbiotics, fecal microbiota transplants and specific dietary interventions have emerged as innovative strategies, increasingly recognised for their documented benefits on mental health.^34^,^37^ Prebiotics—non-digestible substrates that selectively stimulate the growth and activity of beneficial gut bacteria^38^—have been shown to significantly modulate the microbiota-gut-brain axis. These effects include shifts in gut microbial composition, enhanced production of SCFAs, increased circulating levels of brain-derived neurotrophic factor and improvements in cognitive performance and psychological well-being.^3339^,^44^

Given that disruptions in the gut microbiota may exacerbate neuroinflammatory processes and contribute to the progression of neurological disorders, maintaining gut health is essential to preserving brain function and mitigating the impact of alcohol misuse. Furthermore, as the effects of BD on gut microbiota remain a relatively underexplored area, microbiota-targeted interventions may offer a promising means of restoring microbial balance and reducing the neurotoxic consequences of excessive alcohol intake.

### Aims and hypotheses

In light of the evidence presented above, the main objectives of this multidisciplinary study are as follows: (O1) (Binge and Bugs) to evaluate whether a BD pattern of alcohol consumption may lead to gut microbiota alterations in young college students; (O2) (Bugs and Brain) to determine whether the alcohol-induced changes in microbial profile are associated with immune, cognitive, neurostructural and neurofunctional impairments in young BDs; (O3) (Bugs, Brain and Binge) to examine whether the administration of prebiotics (inulin)—through a randomised controlled trial (RCT)—improves gut microbiota composition and, consequently, reduces alcohol-related cognitive and brain impairments. To address these objectives, the following hypotheses will be tested:

H1: Compared with age-matched controls (ie, non/low drinkers), BDs will exhibit disruptions in gut microbiota composition. Specifically, they are expected to show reductions in gut diversity and/or disruptions in bacterial richness (eg, lower levels of *Bacteroidetes* and *Firmicutes*, and increased abundance of *Proteobacteria*).
H2: Alcohol-induced alterations in gut microbiota will be associated with increased levels of peripheral pro-inflammatory markers (eg, interleukin (IL)-1β, IL-6 and tumour necrosis factor-α (TNF-α)).^45^,^47^ Additionally, based on previous literature, the microbiota profile will tentatively be associated with neuropsychological performance,^29^ brain morphometry (eg, amygdala volume^48^) and brain functioning linked to emotional processing and executive control.^49^,^51^
H3: In BDs, prebiotic administration will promote the restoration of gut microbiota composition (primary outcome), as indicated by increased α-diversity and greater bacterial richness compared with pre-intervention assessment. This restoration is expected to mitigate alcohol-induced brain damage in young BDs, with anticipated improvements across various secondary outcome domains, including immunological markers (eg, plasma levels of IL-1β, IL-6, IL-8 and TNF-α), cognitive performance (measured via neuropsychological tasks from the Cambridge Neuropsychological Test Automated Battery (CANTAB)) and neurofunctional and neurostructural indices (eg, resting-state connectivity, task-related brain activity and grey/white matter volumes assessed through MRI).

## Methods and analysis

### Study design and setting

**Figure 1:**
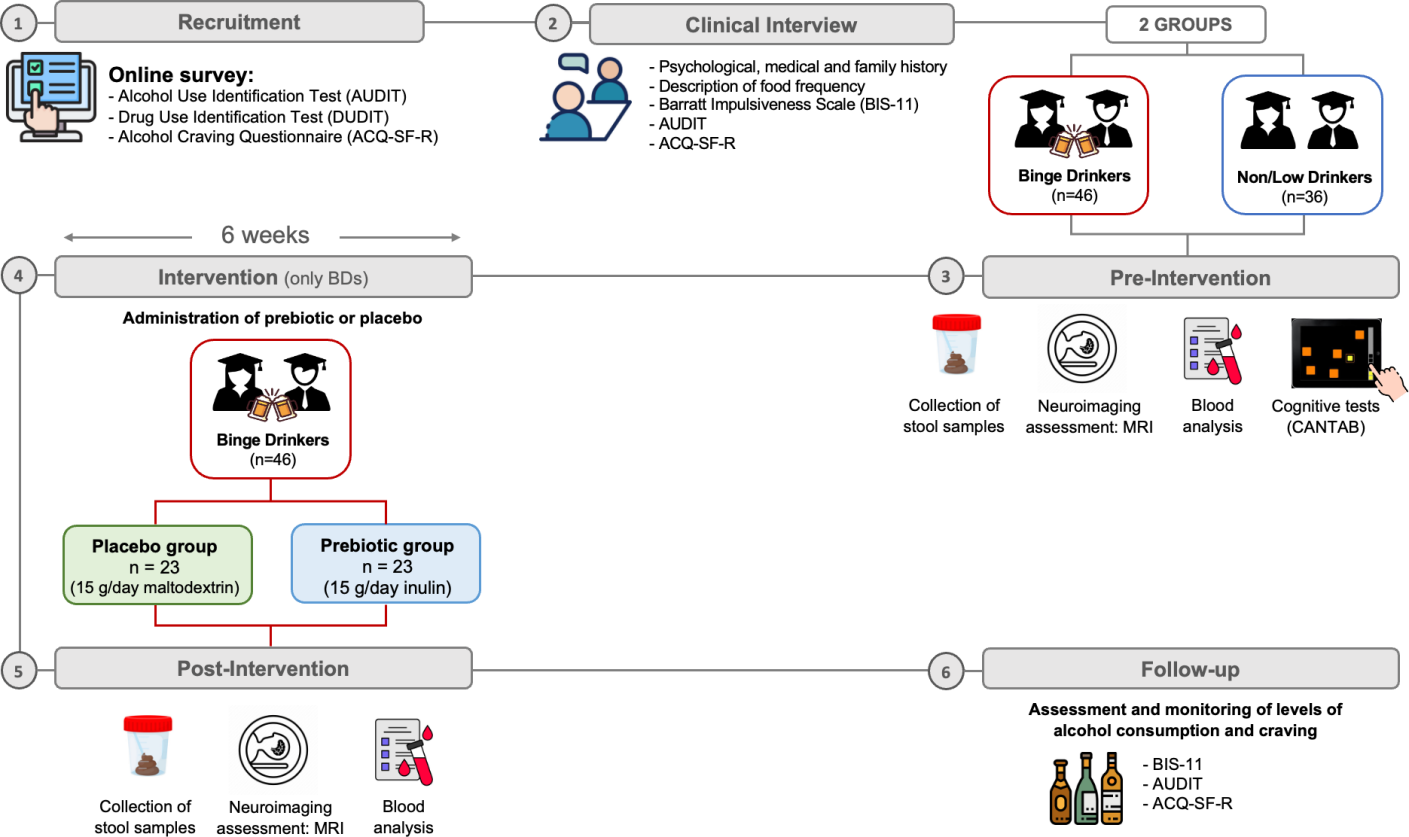
Study outline. 82 participants will be recruited: 36 non/low drinkers and 46 binge drinkers (BDs), matched for age and gender. Recruitment will be carried out through an online survey. After sample selection, participants will undergo a clinical interview covering psychological, medical, personal and family history. According to their drinking patterns, participants will be randomly assigned to two groups: the control group (non/low drinkers) and the intervention group (BDs). During the pre-intervention phase, all participants will be assessed for the variables of interest through neuropsychological tests, collection of stool and blood samples and MRI recordings. Subsequently, only the BDs will proceed to the intervention phase, which involves taking either a prebiotic or a placebo for 6 weeks. After this period, the variables assessed in the pre-intervention phase will be re-evaluated and, over the following 3 months, the levels of alcohol consumption and craving will be monitored.

The present protocol, graphically summarised in figure 1, was registered in the clinical trials database of the National Institutes of Health (NCT05946083). The experimental phase of the study, which included tasks such as randomising the participants and planning the interventions, began in February 2023. While initial dissemination of the study (eg, through institutional email) began in April 2023, the actual participant recruitment, defined as the beginning of clinical interviews for sample selection, did not start until June 2023 and is expected to be completed by July 2025. The study is scheduled for completion in November 2025. The Standard Protocol Items for Randomized Trials checklist, providing a comprehensive overview of the study’s structure and methodology, is available in online supplemental appendix A1.

#### Recruitment

The recruitment will be carried out through an online survey broadcast using the institutional email, which will include items related to alcohol consumption (eg, frequency, intensity and alcohol use). Detailed information on alcohol and drug use will be obtained through several questionnaires: the Alcohol Use Disorders Identification Test (AUDIT^52^), the Drug Use Disorders Identification Test-Extended (DUDIT-E^39^) and the Alcohol Craving Questionnaire - Short Form Revised (ACQ-SF-R^40^). If this approach does not yield the desired number of participants, we will enhance our efforts by conducting face-to-face visits to classrooms, to promote the study directly to young college students and encourage their participation.

#### Clinical interview

A detailed clinical interview will be conducted at the School of Psychology of the University of Minho (EPsi, UM) to assess participants’ clinical history and determine eligibility. The interview will cover psychological, personal, family and clinical history, including and will incorporate standardised instruments related to substance use (eg, AUDIT, ACQ-SF-R), and psychological traits, such as the Symptom Checklist-90-Revised questionnaire (SCL-90-R^41^) and the Barratt Impulsivity Scale (BIS-11^42^). A food frequency questionnaire will also be administered to characterise each participant’s usual eating patterns.^43^ After this stage, and according to their drinking patterns, participants will be assigned to one of two groups: the control group (non/low drinkers) and the BD group.

#### Pre-intervention

The pre-intervention phase involves a comprehensive neuropsychological assessment using tasks from the CANTAB (Cambridge Cognition, UK),^53^ aimed at evaluating participants’ emotional and cognitive abilities (see table 1). These tasks will be administered using a tablet device (iPad) and conducted at the EPsi (UM) on the same day as the clinical interview, with a total duration of approximately 60 min.

**Table 1:** Summary of the cognitive tasks included in the present protocol, selected from the Cambridge Neuropsychological Test Automated Battery (CANTAB)

Task	Cognitive dimensions	Assessed cognitive functions	Duration
*Delayed Matching to Sample (DMS*)	Memory	Visual matching ability; Short-term visual recognition memory.	7 min
*Emotion Recognition Task (ERT*)	Emotion and Social Cognition	Emotional recognition: ability to identify six basic emotions (sadness, happiness, fear, anger, disgust and surprise) in facial expressions.	6 min
*Cambridge Gambling Task (CGT*)	Executive Function	Decision-making; Risk behaviour outside a learning context.	12 min
*Intra-Extra Dimensional Set Shift (IED*)	Executive Function	Cognitive flexibility.	7 min
*Spatial Working Memory (SWM*)	Executive Function	Working memory strategies and capacity.	6 min
*Stop Signal Task (SST*)	Executive Function	Response inhibition/impulse control.	14 min

In order to establish a robust database for comparative analysis, stool and blood samples will be collected to assess gut microbiota diversity/composition, as well as to examine the inflammatory response, respectively. In addition, MRI will be performed to obtain neurostructural and neurofunctional data for each participant (see section 2.4. Assessment protocol, topics 2.4.2. Gut microbiota and 2.4.3. Neuroimaging assessment). All samples and imaging data are expected to be collected within a time frame of 72 hours.

#### Intervention

The BD group will be randomly assigned to one of two intervention subgroups: prebiotic or placebo. Inulin (Frutafit IQ from Sensus B.V.), a well-established prebiotic fibre,^46 47^ will serve as the active intervention. Maltodextrin, which has a taste and appearance similar to inulin but is not expected to impact the regulation of the intestinal microbiome,^44^ will be used as the placebo. Depending on their assigned group, each participant will receive one of two types of fibre over a 6-week period, with a total daily dose of 15 g, divided into three doses per day.^48 49^

To uphold scientific standards and minimise bias, the study will be conducted under a double-blind design with respect to group assignments. Throughout each intervention week, participants in the intervention group will record their dietary intake using a food diary,^43 50^ documenting all food and beverages consumed over 3 days: two weekdays and one weekend day. To promote adherence, each daily dose will be provided in a pre-measured, individually labelled container indicating the date of intake. Participants will receive the intervention in 2-week batches and attend biweekly check-ins with the research team to provide feedback and collect the subsequent set of doses.

#### Post-intervention

The post-intervention phase will begin with a new collection of stool and blood samples, along with MRI scans, aimed at identifying potential changes in the microbiota-gut-brain axis resulting from the intervention.

#### Follow-up

The follow-up phase will extend over 3 months post-intervention, focusing on the assessment and monitoring of alcohol use and craving. During this period, BDs will complete specific questionnaires related to alcohol use (eg, AUDIT, ACQ-SF-R) and psychological symptoms (eg, SCL-90-R).

### Study population

A total of 82 college students (36 non/low drinkers and 46 BDs) from the UM (Braga, Portugal), aged between 18 and 23 years and matched for age and gender, will be recruited for the study. The sample size was determined based on statistical power analysis for two groups (control and BDs), using the G*Power software.^51^ Assuming an α level of 0.05, a power of 0.80 and an effect size of 0.6 (considered a medium effect size), a minimum of 36 participants was required for the control group. For the BD group, the same parameters yielded a required sample size of 19 participants per intervention subgroup (inulin vs maltodextrin). To account for an anticipated dropout of approximately four individuals for each BD subgroup, the adjusted sample size was set to 23 subjects per subgroup, resulting in a total of 46 BDs.

Participants who report (1) consuming five or more drinks on a single occasion at least once per month and (2) drinking at a rate of at least two drinks per hour during these episodes will be classified as BDs. Those who (1) never drank five or more drinks on any occasion and (2) had an AUDIT score ≤4 will be considered non/low drinkers.

The exclusion criteria will be as follows: use of illegal drugs, as determined by the DUDIT-E, except cannabis (ie, at most once a month); alcohol abuse (ie, AUDIT score ≥20); personal history of psychopathological disorders (according to the Diagnostic and Statistical Manual of Mental Disorders (DSM-5) criteria); history of traumatic brain injury or neurological disorders; family history of substance abuse (including alcoholism); any episode of loss of consciousness lasting more than 30 min; non-corrected sensory deficits; presence of metallic implants in the body, particularly in the head region (orthodontic devices are not considered exclusionary); score ≥90 on the Global Severity Index of the SCL-90-R or on at least two of its symptomatic dimensions; and diagnosis of any gut disease/problems or other medical conditions, including inflammatory bowel disease, irritable bowel syndrome, Crohn’s disease, coeliac disease, lactose intolerance and autoimmune disorders. Regarding medicine consumption, individuals will also be excluded if they have used psychoactive medications (eg, antidepressants, benzodiazepines, sedatives or anxiolytics) within 4 weeks prior to the experiment. During the same period, the use of any of the following medical drugs will also result in exclusion: laxatives, antibiotics, anticoagulants, non-steroidal anti-inflammatory drugs, analgesics and corticosteroids. In addition, participants who have consumed any form of prebiotic or probiotic supplements within the month preceding the experiment, as well as those who follow a vegan diet, will not be included.

### Randomisation and blinding

The intervention protocol—comprising either prebiotic or placebo—will be implemented by a qualified research assistant not otherwise involved in the study, who will be responsible for packing the prebiotic and placebo and randomly assigning them to participants in each BD subgroup. Over the course of 6 weeks, BDs will receive a daily dose of 15 g of the assigned intervention (divided into three equal administrations per day: (1) a prebiotic inulin intervention (n=23) or (2) a placebo intervention with maltodextrin (n=23)). Group allocation will remain concealed from both participants and research staff until the completion of the final analyses.

### Assessment protocol

#### Neuropsychological evaluation

During both the pre-intervention phase (for all participants) and post-intervention phase (only for BDs), participants will complete a series of neuropsychological tasks using the CANTAB, a well-validated, computerised test battery widely used in both clinical and research settings that provides a comprehensive evaluation of cognitive functions.^45^ The neurocognitive assessment will focus on executive functions—namely, working memory, inhibitory control, cognitive flexibility and decision-making—as well as short-term memory and emotion recognition abilities (see table 1).

#### Gut microbiota

##### Stool samples

Stool samples will be collected from the control (pre-intervention) and the BD (both pre-intervention and post-intervention) groups for gut microbiota diversity/composition analysis by 16S rRNA metagenomics (Illumina sequencing). Each sample will be collected using the EasySampler Stool Collection Kit, kept under anaerobic conditions and refrigerated until delivery to the laboratory. The sample will be aliquoted at the Centre of Biological Engineering (UM) and stored at −80°C for later analysis. Participants will also be instructed to collect the sample as close as possible to the pre-intervention or post-intervention visit, with a maximum window of 24 hours prior.

Each stool sample will be further subdivided into 1 g aliquots, suspended in sterile 0.1 M phosphate-buffered saline at 10% and stored at −20°C until DNA extraction. Genomic DNA will be extracted and purified using the NZY Tissue gDNA Isolation Kit (NZYtech, Portugal), with minor protocol modifications.^54^ DNA purity and quantification will be evaluated with a NanoDrop spectrophotometer. Furthermore, sequencing of the bacterial 16S rRNA V3-V4 gene region will be performed by RTL Genomics (Lubbock, Texas, USA) using the Illumina MiSeq platform (Illumina Inc., San Diego, California, USA).

To assess the impact of prebiotic administration on gut microbiota, participants in the intervention group will also complete a food frequency questionnaire during the clinical interview and a food diary throughout the intervention. In this diary, subjects will report all food and beverage intake over 3 days per intervention week (two weekdays and one weekend), including details of packaging and location of consumption.

##### Blood samples—inflammatory markers

Blood samples will be collected by a qualified nurse at the EPsi (UM) during both pre-intervention and post-intervention phases. Participants will be instructed to fast at the time of collection. Samples will be stored in BD Vacutainer tubes containing ethylenediamine tetraacetic acid. Subsequently, the plasma will be aliquoted and preserved at –80°C until analysis. Peripheral levels of the interleukins IL-1β, IL-6 and IL-8 and the TNF-α will be determined by ELISA kits.

### Neuroimaging assessment

Neurostructural and neurofunctional assessments will be conducted during the pre-intervention (for all participants) and post-intervention (only for BDs) phases at the Hospital da Luz (Guimarães, Portugal). Scanning will be performed by certified radiology and nursing staff in collaboration with the research team. Participants will be instructed to abstain from consuming alcohol within 24 hours prior to the session and to avoid BD episodes for at least 3 days preceding the MRI session. Before scanning, potential alcohol use will be tested using a breathalyser device, and the assessment will only proceed after confirming 0% breath alcohol level. Furthermore, participants will be asked to refrain from smoking or consuming tea or coffee for at least 3 hours prior to the assessment.

Recordings will be conducted in a 3 T Magneton Trio (Siemens) clinical MRI scanner equipped with an eight-channel receive-only head coil. Participants will wear earmuffs to reduce the scanner noise, and head motion will be minimised using foam pads. The neuroimaging recording protocol will include structural (T1-weighted and diffusion-weighted imaging (DWI)) and functional (T2*-weighted) acquisitions. The total acquisition time will be approximately 57 min, divided into 5.35 min of T1, 14.12 min of DWI, 7.03 min of resting-state fMRI and 30.32 min for task-related acquisitions. The total duration of the experiment (including preparation, acquisition protocol and task performance) will be around 100 min. During task-related fMRI acquisitions, participants will view images of experimental tasks on a large monitor at the opening of the MRI scanner. A mirror mounted on the head coil will allow participants to see the stimuli correctly oriented, despite their being displayed in reverse on the monitor. Behavioural responses will be recorded using MRI-compatible four-button fibre optic response pads (ResponseGrips, Nordic Neurolab). Further details regarding MRI signal acquisition and analyses are provided in online supplemental appendix A2.

#### Experimental tasks (figure 2)

**Figure 2:**
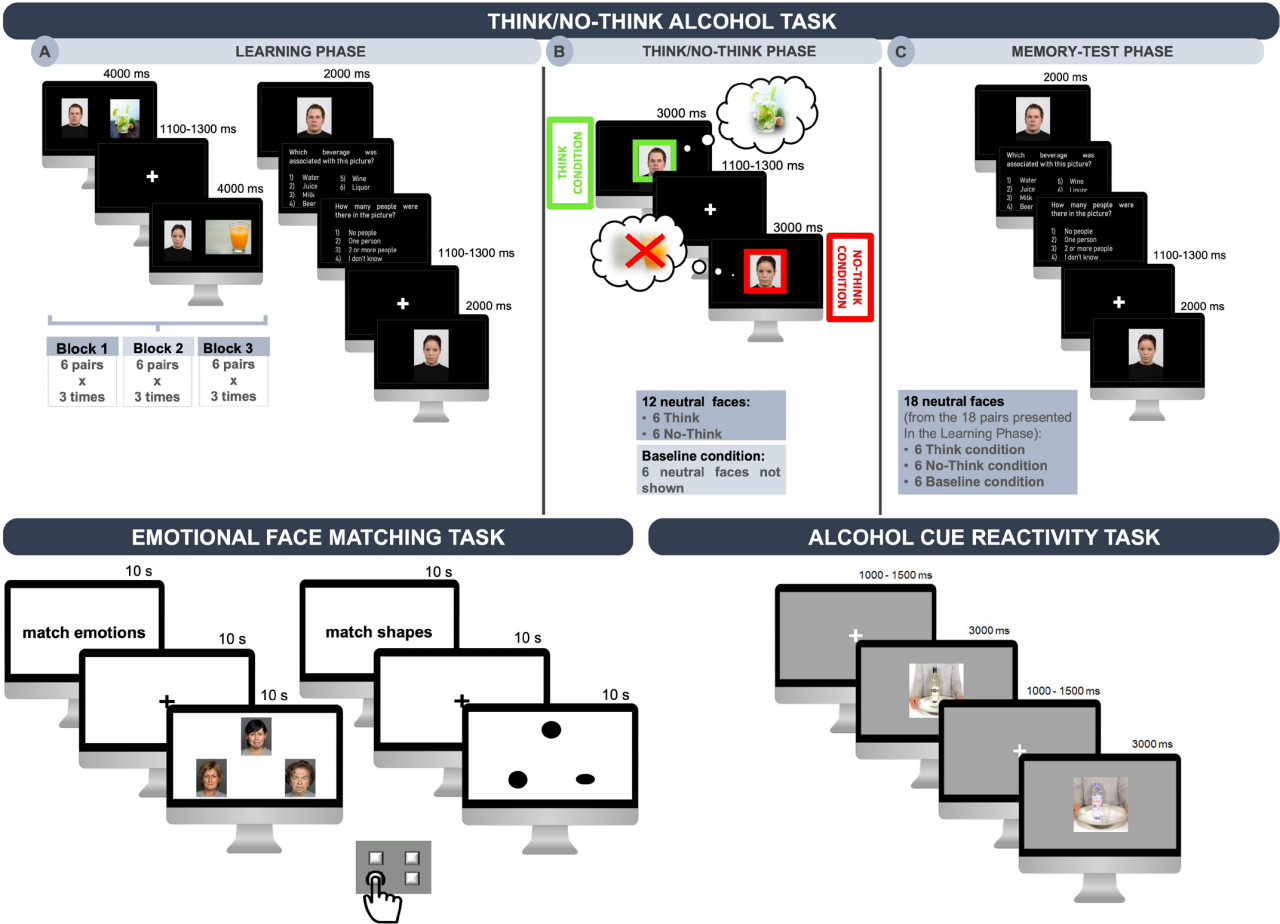
Graphic representation of the three tasks used during brain activity recordings in the MRI scanner: the Think/No-Think Alcohol task (TNTA; top), the Emotional Face Matching task (EFM; bottom left) and the Alcohol Cue Reactivity task (ACR; bottom right). The TNTA task uses beverage images (alcoholic and non-alcoholic) from the Galician Beverage Picture Set^56^ and neutral-expression human faces from the Radboud Faces Database.^57^ In the EFM task, the experimental condition involves faces expressing anger, sadness or fear, sourced from the FACES database*,^59^ while the control condition uses geometric shapes (ovals or circles). The ACR task includes beverage images (alcoholic and non-alcoholic) from the Amsterdam Beverage Picture Set^62^ and neutral objects images from the POPORO database.^63^ *Note: The faces displayed in the EFM task are shown for illustrative purposes only, as they are the publicly available examples permitted for dissemination according to the FACES database guidelines. In the experimental task, all faces were matched for age and gender.

*Think/No-Think Alcohol (TNTA) Task:* The TNTA task assesses memory inhibition mechanisms in alcohol-related contexts.^55^ It uses 18 beverage images (9 alcoholic and nine non-alcoholic) from the Galician Beverage Picture Set,^56^ and 18 neutral-expression human faces (equally distributed across three age groups: young, middle-aged and older) from the Radboud Faces Database.^57^ The beverages are grouped into six types: three alcoholic (beer, wine and liquor) and three non-alcoholic (water, juice and milk). Each pair consists of one neutral face and one drink image. The TNTA task comprises three phases: Learning, Think/No-Think (TNT) and Memory Test. In the Learning phase, participants are asked to memorise 18 image pairs, divided into three blocks of six pairs each. Each block starts with a sequential random presentation of the six pairs on the screen for 4 s. Afterwards, only the human faces are shown, and participants must attempt to recall the associated image (alcoholic or non-alcoholic) by answering two questions: “Which drink was associated with this image?”; “How many people were there in the picture?”. In each block, the six image pairs and the questions are repeated three times. At the end of each block, participants receive feedback on the number of correct responses. An answer is considered correct only if participants respond correctly to both questions. In the TNT phase, only the faces with neutral expressions are shown. Participants are instructed to either *think* (indicated by a green frame surrounding the face)—meaning they should focus on the face and recall the associated alcoholic or non-alcoholic image—or to *no-think* (indicated by a red frame surrounding the face)—meaning they should focus on the face and actively prevent the previously associated picture from entering their consciousness. During this phase, only 12 faces are depicted, as the remaining 6 serve as a baseline condition for the following phase. Finally, in the Memory-Test phase, all 18 neutral faces from the initial pairs are presented again, and participants are asked to remember the image (alcoholic or non-alcoholic) that was originally associated with each face, as indicated by the two questions from the Learning phase.

*Emotional Face Matching (EFM) task:* The ability to recognise emotions will be assessed through the EFM task.^58^ This paradigm measures both preconscious and conscious brain responses to emotional stimuli by contrasting an experimental condition (emotional face matching) with a control condition (shape matching). The experimental condition involves faces expressing anger, sadness or fear, sourced from the FACES database,^59^ while the control condition uses geometric shapes (ovals or circles). The task comprises six blocks—three emotion-matching and three shape-matching—presented in alternating order without breaks. The total duration is approximately 10 min. Each block consists of 18 trials, each lasting 5 s with no interstimulus interval. At the beginning of each block, instructions (‘match emotions’ or ‘match shapes’) are presented for 10 s, followed by a 10 s fixation cross. In each trial, three stimuli are displayed in a triangular arrangement: one stimulus (face or shape) appears at the top of the screen and two at the bottom. Participants are instructed to match the top stimulus with one of the two bottom stimuli based on emotional expression or shape by pressing the corresponding button on a handheld MRI-compatible response device.

*Alcohol Cue Reactivity (ACR) task:* Reactivity to alcohol-related cues will be assessed using the ACR task.^60^ This task starts with a white fixation cross displayed on a grey background for a variable duration ranging from 100 to 150 ms. Subsequently, either an alcoholic or a non-alcoholic drink is randomly presented at the centre of the screen for 300 ms. Participants are instructed to attentively observe all images presented on the screen. Similar to the study by Wiers *et al*,^61^ designed to assess participants’ task engagement, four oddball blocks were included. Each oddball block consists of four alcoholic or non-alcoholic stimuli interspersed with a single oddball cue, represented by a neutral object (eg, a button or a padlock). During these blocks, participants are required to press a button with their right hand upon seeing the oddball image. After completing the image viewing, participants are asked to rate their emotional responses to the alcoholic or non-alcoholic beverage images in terms of valence, arousal and craving/desire, using the Self-Assessment Manikin scale.^59^ The task includes a total of 80 beverage images (40 alcoholic and 40 non-alcoholic), sourced from the Amsterdam Beverage Picture Set^62^ and neutral objects images from the POPORO database.^63^

### Statistical analysis plan

The main statistical analyses will focus on comparisons between BDs and non-/low-drinking controls across the different outcome domains. To account for potential confounding effects, sociodemographic variables such as sex and age will be included as covariates in all relevant models—including microbiota, immune, cognitive and imaging analyses—ensuring appropriate control of their influence on the primary latent variables.

Regarding statistical analysis for gut microbiota, the primary outcome will focus on assessing between-group differences at two levels: α-diversity (within-sample) and β-diversity (between-sample).^64^ Measures of *α*-diversity will include the following: (1) Species richness (total number of differential abundance of microbial species); (2) Chao1 index (a nonparametric richness estimator); (3) Shannon diversity index (a metric combining richness and evenness, assigning equal weight to abundant and rare species); and (4) Simpson diversity index (also accounting for richness and evenness, but giving more weight to more abundant species). Measures of β‐diversity—that is, the similarity or distance between microbiome pairs—will include Bray–Curtis dissimilarity (a measure of community composition differences), and weighted and unweighted UniFrac (indices of phylogenetic community differences).

Quantification data will be normalised by the mean number of 16S rRNA gene copies specific to each detected microorganism. Microbial community analysis will be performed by sequencing the 16S rRNA gene (V4 region) using the prokaryotic universal primer pair 515f/806r,^65^ by MiSeq Illumina sequencing at Research and Testing Laboratory (Lubbock, Texas).^66^ Detailed information on the bioinformatics analysis steps can be found on the RTL website (https://rtlgenomics.com/amplicon-bioinformatics-pipeline). Microbiota composition data (with a focus on dominant phyla such as *Firmicutes*, *Bacteroides*, *Actinobacteria* and *Proteobacteria*) will be correlated with data obtained at the beginning and end of the intervention. In line with open science policies, sequencing data (FASTQ files) will be submitted to a publicly accessible repository, such as the European Nucleotide Archive of the European Bioinformatics Institute.

The intervention is expected to promote the restoration of gut microbiota composition in young BDs, which will be assessed through secondary outcomes. These outcomes are anticipated to reflect improvements across multiple domains: (1) immunological, through reductions in pro-inflammatory markers; (2) cognitive, via enhanced neuropsychological performance; and (3) neurofunctional/neurostructural, for instance, evidenced by decreased neural reactivity to alcohol-related cues.

In particular, to evaluate immunological changes, the study will characterise the levels of key inflammatory markers—including the TNF-α and the interleukins IL-1β, IL-6 and IL-8—in the blood of individuals with BD in comparison with the control group. Normality of the data will be tested using the Shapiro-Wilk test. For normally distributed variables, comparisons between groups (non/low drinkers vs BDs, and inulin vs maltodextrin interventions) will be performed using the independent samples t-test. For non-normally distributed data, the Mann-Whitney U test will be applied. Correlations between inflammatory biomarkers and other relevant variables will be assessed using Pearson’s or Spearman’s correlation coefficients, depending on the distribution. A significant level of 0.05 will be adopted for all statistical tests.

Neuropsychological performance will be analysed using the key outcome measures defined for each task (see online supplemental appendix A3), including, but not limited to, the number of correct responses, mean reaction times and commission and omission errors. Following the same approach as for the analysis of pro-inflammatory markers, independent samples t-tests will be conducted to examine potential mean differences between BDs and non/low drinkers.

For structural and resting-state fMRI data, multivariate analysis of variance and two-way mixed ANOVAs will be applied with group (non/low drinkers vs BDs) and sex (male vs female) as the between-subjects factors, hemisphere (left vs right) as the within-subjects factor, and age as a covariate to test for differences in grey and white matter volumes, functional connectivity and resting-state activity. For these and the remaining analyses, df will be corrected by the Greenhouse-Geisser procedure when appropriate. Family-wise error (FWE) corrections will be applied to both main and interaction effects, and post hoc paired comparisons will be conducted with the Bonferroni adjustment for multiple comparisons (α≤0.05). Regarding task-related brain activity, the contrast images from the first-level analysis will be used for the second-level analysis. We will analyse the data using a repeated measures ANOVA for each task to assess the main effects of group (ie, non/low drinkers vs BDs), conditions (eg, alcohol vs non-alcohol, think vs no-think), and the group × conditions interactions using the flexible factorial approach in SPM12. A whole-brain cluster FWE correction at p<0.05 will be applied, with an uncorrected voxel-level cluster-defining threshold of p<0.001.

To explore the complex relationships among alcohol use, microbial diversity/composition, neuropsychological measures, pro-inflammatory markers and structural and functional brain characteristics, we will develop a Structural Equation Modeling (SEM).^67^ This model will encompass six primary latent variables: Alcohol Use Patterns, Gut Microbiota, Neuropsychological Performance, Immune Response, Brain Structure and Brain Functioning. Each of these latent variables will be operationalised by the aforementioned observed variables (eg, membership in the BD or non/low drinker group, α-/β‐diversity, levels of TNF-α and interleukins, volumes of grey and white matter). In the proposed SEM model, Alcohol Use Patterns are conceptualised as an exogenous latent variable, reflecting individual differences in drinking behaviour that are hypothesised to influence gut microbiota composition and inflammatory processes. Gut Microbiota and Immune Response are modelled as mediators through which alcohol-related effects may impact brain structure and function, as well as cognitive performance. This formulation is grounded in previous findings highlighting the role of chronic alcohol use in inducing gut dysbiosis and systemic inflammation,^68^ which may contribute to neuroinflammatory processes and cognitive impairment.^12 28^ Additionally, covariances between latent variables will be examined to elucidate interactions among the various dimensions of the study. Sex and age will also be included in the SEM as covariates to account for their potential influence on the relationships among latent variables.

Finally, the RCT will aim to determine whether prebiotic administration in a subgroup of BDs can affect the microbiota diversity/composition and if these changes may influence other health measures such as inflammatory response or brain volume. To this end, we will employ a mixed-effects linear regression model to assess how treatment (prebiotic vs placebo) influences outcomes related to immune response, neuropsychological performance and brain structure and function in a sample of individuals characterised by BD behaviour.

### Patient and public involvement

Due to the specific nature of our research and its methodological constraints, it was neither appropriate nor feasible to involve patients or the public in the design, conduct, reporting or dissemination plans of this study. Consequently, the research was conducted without direct input from patients or public representatives.

## Ethics and dissemination

The Ethics Committee for Social and Human Sciences of the University of Minho approved the present protocol on 28 June 2022 (approval reference: CEICSH 078/2022). All research procedures will be conducted in accordance with national regulations and internationally accepted ethical standards, including the Declaration of Helsinki (64th World Medical Association General Assembly, Brazil, 2013) and the Code of Ethical Principles for Medical Research Involving Human Subjects.

All data will be treated confidentially and will be used exclusively for research purposes by the research team, in compliance with the General Data Protection Regulations (GDPR). Researchers will underscore that participation is voluntary and that individuals can withdraw from the study at any time. Before enrolling in the study, participants will be informed about the aims, conditions and procedures of the study and provided with two copies of the informed consent form (see online supplemental appendix A4), one for the researchers and one for the participants. College students will receive gift vouchers to compensate for their participation.

Ethical issues identified are related to the collection and processing of sensitive personal health/lifestyle data from participants. All procedures employed in the study are non-invasive and considered safe. The prebiotics administered in this randomised clinical trial have been shown to have no known adverse effects.^69^ They are classified as food ingredients in Europe,^70^ hold Generally Recognized as Safe status in the USA^71^ and are recognised as dietary fibres by the Food and Drug Administration.^72^

Following a thorough analysis of the project data, the findings will be disseminated regardless of the outcome. Findings will be presented at national and international conferences and published in peer-reviewed scientific journals indexed in the Journal Citation Reports, preferably in open-access formats. Additionally, data may be shared with third parties solely for research purposes and upon reasonable request, in line with ethical guidelines and data protection regulations. The anonymity of all participants will be strictly maintained, and all data will be handled in compliance with relevant data protection laws, including the GDPR.

## Discussion

To the best of our knowledge, this protocol will be the first to explore the potential relationship between the *Brain* (neurocognitive functioning), the *Bugs* (gut microbiota) and the *Binge* (alcohol use) in a population of youths with a BD pattern in comparison with youths with low or no alcohol use. This study will also implement an RCT aimed at exploring the potential effects of a prebiotic intervention along the microbiota-gut-brain axis in this population.

The uniqueness of this protocol lies in the exploration of the complex interaction between alcohol, brain activity and gut microbiome through the implementation of multiple levels of analysis, including techniques to measure brain activity (ie, MRI), paradigms to measure cognitive performance, as well as stool and blood sampling, and the application of various questionnaires concerning psychological and behavioural characteristics. By integrating these diverse methodologies, we aim to elucidate the intricate mechanisms underlying the effects of alcohol consumption on both the brain and the gut microbiome, shedding light on potential biomarkers and cognitive changes associated with different levels of alcohol intake. This comprehensive approach not only allows for a more holistic understanding of the impact of alcohol on human physiology and cognition but also opens up new avenues for tailored interventions and preventive strategies aimed at mitigating the negative consequences of excessive alcohol consumption.

Despite the methodological robustness and the proposed multidimensional approach, this study presents some limitations that should be acknowledged. First, participant adherence, particularly among young binge drinkers, may prove challenging, as the extended duration of the intervention (6 weeks) and the level of commitment required could hinder sustained participation and, consequently, compromise the validity of the collected data. Additionally, several external factors, such as exercise, sleep patterns and stress levels, will not be strictly controlled, which may introduce significant variability and confound the observed effects of the intervention on the microbiota-gut-brain axis. Logistical challenges related to the collection of biological samples and neuroimaging data, such as scheduling difficulties, lack of transportation or participant discomfort, may increase the risk of missing data or dropout. Finally, interruption of the treatment during the intervention phase, motivated by adverse effects, forgetfulness or low motivation, may also compromise the consistency of the intervention. To mitigate the impact of these limitations, a contingency and risk analysis plan was drawn up, presented in online supplemental appendix A5, which outlines potential eventualities throughout the study as well as proposed strategies for their mitigation.

In summary, this protocol outlines a novel and integrative approach to investigate the relationship between alcohol consumption, brain function and gut microbiota in young college students, combining multiple methodologies and levels of analysis. Despite the identified limitations, the study holds considerable potential to advance scientific understanding in this field, providing a solid foundation for future research and targeted interventions.

## Supplementary material

10.1136/bmjopen-2024-095932 online supplemental file 1

10.1136/bmjopen-2024-095932 online supplemental file 2

10.1136/bmjopen-2024-095932 online supplemental file 3

10.1136/bmjopen-2024-095932 online supplemental file 4

10.1136/bmjopen-2024-095932 online supplemental file 5

## Data Availability

All data relevant to the study are included in the article or uploaded as supplementary information.
