# Supplementary material for: Assessing the impact of binge drinking and a prebiotic intervention on the gut–brain axis in young adults: protocol for a randomised controlled trial
Source: BMJ Open. 2025 Sep 4;15(9):e095932. doi: 10.1136/bmjopen-2024-095932 (PMC12414231; doi:10.1136/bmjopen-2024-095932)
Supplement: online supplemental file 2 [file bmjopen-15-9-s002.docx]

**SUPPLEMENTARY MATERIAL** - **Appendix A2**

1. **MRI acquisition**

Sagittal high-resolution 3D T1 weighted anatomical images will be acquired using a magnetization prepared rapid acquisition gradient echo (MPRAGE) sequence with the following parameters: repetition time (TR) = 2400 ms, echo time (TE) = 2.53 ms, inversion time (TI) = 1.100 ms, flip angle (FA) = 9°, 176 slices with 0.98 mm thickness, slab thickness = 172.48 mm, slice gap = 0 mm, in-plane resolution = 1x1 mm^2^, matrix size = 256x256 and 250mm FoV.

The Diffusion Weighted Imaging (DWI) will be performed using a spin-echo echo-planar imaging (SE-EPI) sequence: TR = 6200 ms; TE = 96 ms; FoV read=220 mm; acquisition matrix = 91×110; 44 slices, slice thickness = 3.5 mm; 64 non-collinear gradient directions with b = 1000 s/mm2; and two b = 0 s/mm2 acquisition, slice gap = 0 mm, voxel size = 2.4x2x3.5 mm.

The blood oxygen level-dependent (BOLD) sensitive echo-planar imaging (EPI) will be used with the following acquisition parameters: 48 interleaved axial slices; TR = 2000ms; TE = 18ms; FA = 85º; slice thickness = 3mm; voxel size = 4.1x3x3mm; FoV = 200mm. matrix size: 49x66, slice gap = 0 mm.

The resting state fMRI will be performed using a blood oxygen level dependent (BOLD) sensitive echo-planar imaging (EPI) with the parameters: 46 interleaved axial slices, TR = 1460 ms, TE = 12 ms, flip angle (FA) = 85°, slice thickness = 3 mm, voxel size = 5.1x3x3 mm, FoV = 200 mm and 285 volumes.

1. **MRI processing and analysis**

After the acquisition, all MRI scans will be visually inspected to discard critical head motion or brain lesions. After this verification, each subject’s T1-weighted MRI will be processed using the automated reconall FreeSurfer preprocessing, parcellation, and segmentation standard steps (http://surfer.nmr.mgh.harvard.edu) to obtain the cortical surface reconstruction and tissue-class segmentation. Briefly, the pipeline will include motion correction and intensity normalization, removal of non-brain tissue and skull stripping, Talairach transformations, whole-brain segmentation, cortical parcellation, and statistical outputs.

DTI preprocessing will be performed with tools provided by the FMRIB Software Library (FSL- http://fsl. fmrib.ox.ac.uk/fsl/). The analysis pipeline will be carried out as follows: i) motion and eddy current corrections using FMRIB's Diffusion Toolbox (FDT); ii) brain extraction tool (BET) applied to the T1 and DWI images; iii) tensor fitting computation will be performed with DTIFIT; iv) after tensor estimation, tractography and scalar maps of FA, AD, RD and MD will be generated.

Functional imaging data preprocessing and analysis will be performed with Statistical Parametric Mapping SPM12 (Wellcome Trust Centre for Neuroimaging, London, UK). In the preprocessing, all functional images will be corrected for slice timing and motions using realign, co-registered to T1-weight structural image and spatially normalized into the Montreal Neurological Institute (MNI) template. These normalized images will be spatially smoothed using a Gaussian kernel of 8-mm full width at half maximum (FWHM) and low-frequency drifts will be removed with a high-pass temporal filter (filter width of 128s).
