# Supplementary material for: Assessing the impact of binge drinking and a prebiotic intervention on the gut–brain axis in young adults: protocol for a randomised controlled trial
Source: BMJ Open. 2025 Sep 4;15(9):e095932. doi: 10.1136/bmjopen-2024-095932 (PMC12414231; doi:10.1136/bmjopen-2024-095932)
Supplement: online supplemental file 3 [file bmjopen-15-9-s003.docx]

**SUPPLEMENTARY MATERIAL** - **Appendix A3**

1. **CANTAB Connect Research Outcome Measures**

Six tasks of the CANTAB will be administered to evaluate different components of cognitive functions, and task-specific performance measures related to speed as well as task performance accuracy will be analysed to provide a comprehensive profile of cognitive function. Table 1 describes the outcome measures used in the analysis of each task.

**Table 1.** Outcome measures descriptions of the cognitive tasks from the Cambridge Neuropsychological Tests Automated Battery (CANTAB).

| Measure Code | Description |
| --- | --- |
| *Delayed Matching to Sample* | |
| DMSPCAD  *Percent Correct All Delays* | Percentage of success in trials in which the subject chose the correct pattern on the first attempt. Calculated for all trials. |
| DMSPCS, DMSPCO, DMSPC4, DMSPC12  *Percent Correct Simultaneous/0/4/12* | Percentage of success calculated in all trials in which there was an interval of zero, four and twelve seconds between stimulus and response hypotheses. |
| *Emotion Recognition Task* | |
| ERTCRT H/S/F/A/D/SU  *Median Correct Reaction Time* | Reaction time of the correct choice of emotion after being presented with a face, calculated in all tests evaluated. |
| ERTUHR H/S/F/A/D/SU  *Unbiased Hit Rate* | Unbiased hit rate, which ensures that the accuracy of emotion recognition is not influenced by response guessing or response bias effects. |
| ERTTHH/S/F/A/D/SU  *Total Hits* | The number of times the subject correctly selected the emotion in all trials evaluated. |
| ERTTFAH S/F/A/D/SU  *Total False Alarms* | The number of times the subject has incorrectly selected an emotion (false alarms). |
| *Cambridge Gambling Task* | |
| CGTDMQMT  *Decision Making Quality Total Merged* | The ratio (0 – 1) of trials in which the subject chose the colour of the majority squares, calculated for all trials (up and down) in which the number of squares of each colour differed. |
| CGTRAJTM  *Risk Adjustment Merged* | Measure of risk adjustment calculated from the average proportion of points that the subject has chosen to bet, taking into account the majority of coloured squares. |
| CGTDAVT  *Delay Aversion Total* | A measure that allows the dissociation between risk taking and impulsiveness, determining whether the subject made a bet at the first opportunity. Calculated based on the subtraction of risk taking in all ascending trials to risk taking in descending trials. |
| *Intra-Extra Dimensional Set Shift* | |
| IEDEEDS  *EDS Errors* | The number of times that the subject failed to select the rule-compatible stimulus in the phase in which the extradimensional displacement occurs. A measure of the subject’s ability to change the focus of attention. |
| IEDYERTA  *Total Errors Adjusted* | The number of times the subject chose a wrong stimulus (incompatible with the current rule, adjusted for each stage of the task that was not reached). |
| *Spatial Working Memory* | |
| SWMS  *Strategy* | The number of times the subject starts new research. It provides information about strategy. |
| SWMBE468  *Between Errors* | The number of times the subject incorrectly revisits a square in which a token was previously found. Calculated on all trials evaluated (with four, six, and eight tokens). |
| SWMBE4 /SWMBE6 /SWMBE8  *Between Errors 4/6/8 Boxes* | The number of times the subject revisits a square in which a token was previously found. Calculated for each type of test (4, 6, or 8 tokens). |
| *Stop Signal Task* | |
| SSTSSRT  *Reaction Time* | Reaction time for trials in which the participant successfully inhibited the response. |

**Note**: H= Happiness; F= Fear; S= Sadness; A = Anger; D = Disgust; SU = Surprise; DMS = Delayed Matching to Sample; SWM = Spatial Working Memory; ERT = Emotional Recognition Task; IED = Intra-Extra Dimensional Set Shift; SST = Stop Signal Task; CGT = Cambridge Gambling Task.
