# Supplementary material for: Assessing the impact of binge drinking and a prebiotic intervention on the gut–brain axis in young adults: protocol for a randomised controlled trial
Source: BMJ Open. 2025 Sep 4;15(9):e095932. doi: 10.1136/bmjopen-2024-095932 (PMC12414231; doi:10.1136/bmjopen-2024-095932)
Supplement: online supplemental file 4 [file bmjopen-15-9-s004.docx]

**SUPPLEMENTARY MATERIAL** - **Appendix A4**

Informative Document and Informed Consent Forms

**Official Title of the Study:**

Gut Microbiota, Brain Function, and Alcohol Use: Protocol for a Randomised Controlled Trial in Young Adults with a Binge Drinking Pattern

**NCT ID:** NCT05946083


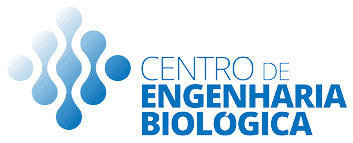

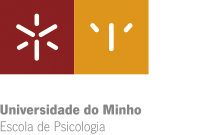


University of Minho

School of Psychology

Research Center in Psychology (CIPsi)

Psychological Neuroscience Laboratory

Campus de Gualtar

4710-057 Braga

Tel: +351 253 601 398

**Research Project reference**: PTDC/PSI-ESP/1243/2021

**Principal Investigator (PI) and Research Team**: Dr. Eduardo López Caned (PI); Dra. Clarisse Nobre

**INFORMATIVE DOCUMENT**

This research project aims to determine the interaction between alcohol consumption, brain function, and gut microbiota through various levels of analysis, including techniques to measure brain activity (i.e. MRI), paradigms to measure cognitive performance, faecal and blood samples, and questionnaires. In addition, this study will investigate the relationship between alcohol, brain activity, and gut microbiota and how this can be modified through our diet.

Thus, the complete evaluation protocol consists of several parts:

1. Interview: this will deal with questions relating to psychological, medical, personal, and family history, including questions alluding to the history of alcohol and drug consumption and some specific questionnaires regarding the use of these substances, as well as relating to physical and psychological symptoms, and personality.
2. Neuroimaging assessment: this will consist of a structural and functional magnetic resonance imaging (MRI) scan at Hospital da Luz (Guimarães, Portugal), while different cognitive tasks are carried out.
3. Assessment of some of the bacteria and microorganisms that reside in the gut: at a certain point in the study, the participant will be asked to collect faeces as well as small blood samples.
4. Evaluation of the potential of an intervention with a prebiotic.

This study will involve the following phases:

1. Pre-intervention, consisting of assessing variables of interest to the study through an interview, carrying out neuropsychological and MRI tests, and collecting faeces and blood;
2. Intervention* (*only for some of the participants), consisting of taking a prebiotic for 6 weeks. Depending on the group to which they will be allocated, the participant will take one of two types of fiber: a fiber with benefits for gut bacteria or a similar fiber without specific benefits for the gut microbiome. Each participant will not know which group they belong to so as not to bias the results of the study according to scientific standards. Both types of fiber are clinically safe (not associated with any health risks);
3. Post-intervention, which will consist of re-assessing the variables previously evaluated in the pre-intervention phase;
4. Follow-up, consisting of assessing and monitoring levels of alcohol consumption and craving during the 6 months following the intervention phase.

To participate, the participant must collaborate positively in the study, answering the questions asked in the interview truthfully and sincerely, and carrying out the tasks, tests, and data collection with a willingness to collaborate with the research team. To achieve the project's objectives, it will be necessary to assess people who fit a certain pattern of alcohol consumption and meet certain criteria for inclusion/exclusion after the participant has been selected based on completing a simple questionnaire. In this sense, after deliberating whether the participant meets the criteria for inclusion in this project, they will be asked to collect demographic and lifestyle data (e.g. age and education, pattern of alcohol consumption and diet, etc.), complete some questionnaires and collect neuroimaging/magnetic resonance imaging data to measure the structure (anatomy) and function (neural activity) of the brain while the participant performs the task, and faecal and blood samples.

The interview will last approximately 60 minutes and will take place in a single session (note: the interview may be split into two at the participant's request). The neuroimaging examinations and pre- and post-intervention collections will be carried out at Hospital da Luz (Guimarães, Portugal) by specialized radiology and nursing staff in collaboration with the research team. The duration of the complete session, which includes the structural and functional neuroimaging examination (including prior preparation), as well as blood sampling, is approximately 2 hours and 30 minutes. It does not require any preparatory tests, medication, or diet. Before entering the scanner room, you will need to fill in a form confirming that you have none of the impediments to the test. As the scanner uses a powerful magnet to record images, it will be necessary to check that you don't have any kind of metal object implanted in your body. In addition, before entering the room where the scanner is located, all metal objects that the participant is carrying with them (i.e. jewelry, piercings, dental plaque) must be removed, as they can distort the images to be obtained in the test. It is therefore advisable to wear clothes that do not have metal pins, otherwise, a hospital gown will be provided.

The technique of magnetic resonance imaging consists of recording cerebral hemodynamic activity. It is a non-invasive procedure (it does not use ionizing radiation - X-rays - or chemical substances) that uses magnets and powerful radio waves to produce images of brain tissue.

None of the tests are painful or produce any side effects whatsoever. The only possible discomfort comes from the restriction of movement and the fact that you must remove metal elements that may be on clothing and accessories. If you are afraid of enclosed spaces, the environment of the MRI machine may not be suitable for you because it is not very open. All these issues are checked by us before we accept your participation in the study through safety questionnaires. The phlebotomy procedure - blood sampling - is a standard procedure that is typically adopted when taking samples for analysis purposes. The venepuncture associated with this procedure may be associated with some localized discomfort or pain at the site of the blood draw. To minimize these effects, alcohol wipes, gauze sponges, adhesive pads and tape will be provided for use after collection. As far as faeces collection is concerned, there is no associated discomfort or risk - it is a typical procedure in routine clinical assessments.

Following the General Data Protection Regulation (EU 2016/679), all personal information collected during the study will be confidential and will not be disclosed to anyone outside the research. Thus, all digital data will be stored on password-protected computers, and in no case will this data contain information identifying the person. Each participant will be assigned an indecipherable code that prevents the data from being associated with the individual. The individual data will be analyzed as part of a group of data on brain, intestinal, and immune activity, among others, obtained from anonymous volunteer participants, and only authorized research staff will have access to the codes that associate the data with personal information. The analysis of the intestinal microbiota will be carried out in collaboration with a clinical laboratory under the same confidentiality and anonymity requirements. Furthermore, during the study, the recommendations of the Declaration of Helsinki and the World Health Organization regarding the development of scientific studies involving human beings will be respected. Participation in this study is voluntary. It is therefore made clear that the participant can decide at any time to stop taking part in the study without this decision giving rise to any consequences.

We will reward participation in the pre-intervention phase with € 30 in vouchers and the intervention and post-intervention phases with € 50 in vouchers, with the participant being rewarded with up to € 80. We will also provide the participant with magnetic resonance images of the brain that may be of medical/diagnostic use in the future if they request them from the researcher.

The participant will also be able to receive an individualized report of their relevant data, whenever requested, but this will not be for diagnostic purposes. However, you will be informed by the team, free of charge, if any neurological problems are detected after the MRI images have been carefully analyzed by the research team.

All data collected may only be used for research purposes and may result in scientific publications and presentations at conferences. However, the results will always be treated anonymously, confidentially, and as a group (i.e. never disclosed individually). The data collected will be subject to a process of anonymization so as not to make the participants identifiable while the data is being processed. All data obtained at Hospital da Luz will be analyzed and stored at the School of Psychology of the University of Minho under appropriate conditions and anonymously. The personal data collected as part of this study will be kept until the final publication of its results, and will not exceed 5 years, after which they will be deleted from the locations where they were stored by the researcher in charge (Dr. Eduardo López-Caneda).

Data subjects are guaranteed the right to access, update, and rectify their data by contacting us directly (+351 253604610 or +351 253601397) or by writing to the principal investigator ([eduardo.lopez@psi.uminho.pt](mailto:eduardo.lopez@psi.uminho.pt)).

Under the terms of the law, you are guaranteed the right to withdraw your consent to the processing of data for the aforementioned purposes through a written request addressed to the principal investigator, which does not, however, invalidate the processing of data carried out up to that date based on the consent previously given.

If you wish to notify us of any aspect relating to the protection of your data, you should do so in writing to the Data Protection Officer at the University of Minho ([protecaodados@uminho.pt](mailto:protecaodados@uminho.pt); <https://www.uminho.pt/protecaodados>) and to the national supervisory authority - the National Data Protection Commission ([www.cnpd.pt](http://www.cnpd.pt)). Thank you in advance for your availability and co-operation.

Eduardo López-Caneda

(Principal Investigator)

Contact (e-mail): [eduardo.lopez@psi.uminho.pt](mailto:eduardo.lopez@psi.uminho.pt)


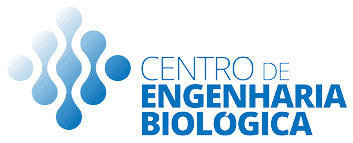

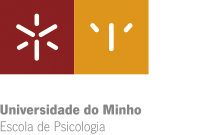


University of Minho

School of Psychology

Research Center in Psychology (CIPsi)

Psychological Neuroscience Laboratory

Campus de Gualtar

4710-057 Braga

Tel: +351 253 601 398

**Research Project reference**: PTDC/PSI-ESP/1243/2021

**Principal Investigator (PI) and Research Team**: Dr. Eduardo López Caned (PI); Dra. Clarisse Nobre

# INFORMED CONSENT FORM

**COPY FOR THE PARTICIPANT**

1. I confirm I have read and understood the informative document that was delivered to me, with all the information regarding the study in which I am participanting, and that I had the opportunity to raise questions and doubts about it.
2. I confirm the research team had provide me clear answers to all my questions and doubts.
3. I understand that I am free to leave the study at every moment, without justification, and without any consequences.
4. I understand and agree that my personal identification data and the data obtained trhough the course of the research study will be kept in separate archives, therefore guaranteeing its safety, and that the team members with access to the data will respect their confidentiality.
5. I, therefore, consint that my data are stored and/or exported to external databases in order to be analyzed, understading that, in any circumstance, information regarding my identity will not be disclosed.
6. I understand that the presente study does not have a diagnostic purpose and, consequentely, I will not receive na individual report with my data/results.
7. I consint to participate in the abovemnetioned study.

**Participant name**: .........................................................................................................................

**Researcher name:** .........................................................................................................................

**Name of the person responsible for collecting this consent** (if different from the researcher):

.........................................................................................................................................................

**Date**: ..............................  **Participant Code**: .................................................


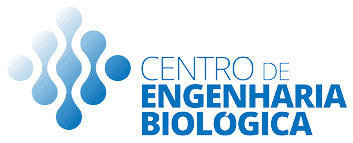

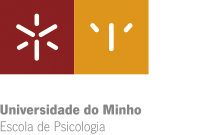


University of Minho

School of Psychology

Research Center in Psychology (CIPsi)

Psychological Neuroscience Laboratory

Campus de Gualtar

4710-057 Braga

Tel: +351 253 601 398

**Research Project reference**: PTDC/PSI-ESP/1243/2021

**Principal Investigator (PI) and Research Team**: Dr. Eduardo López Caned (PI); Dra. Clarisse Nobre

# INFORMED CONSENT FORM

**COPY FOR THE RESEARCHER**

1. I confirm I have read and understood the informative document that was delivered to me, with all the information regarding the study in which I am participanting, and that I had the opportunity to raise questions and doubts about it.
2. I confirm the research team had provide me clear answers to all my questions and doubts.
3. I understand that I am free to leave the study at every moment, without justification, and without any consequences.
4. I understand and agree that my personal identification data and the data obtained trhough the course of the research study will be kept in separate archives, therefore guaranteeing its safety, and that the team members with access to the data will respect their confidentiality.
5. I, therefore, consint that my data are stored and/or exported to external databases in order to be analyzed, understading that, in any circumstance, information regarding my identity will not be disclosed.
6. I understand that the presente study does not have a diagnostic purpose and, consequentely, I will not receive na individual report with my data/results.
7. I consint to participate in the abovemnetioned study.

**Participant name**: .........................................................................................................................

**Researcher name:** .........................................................................................................................

**Name of the person responsible for collecting this consent** (if different from the researcher):

.........................................................................................................................................................

**Date**: ..............................  **Participant Code**: .................................................
