# Supplementary material for: Assessing the impact of binge drinking and a prebiotic intervention on the gut–brain axis in young adults: protocol for a randomised controlled trial
Source: BMJ Open. 2025 Sep 4;15(9):e095932. doi: 10.1136/bmjopen-2024-095932 (PMC12414231; doi:10.1136/bmjopen-2024-095932)
Supplement: online supplemental file 5 [file bmjopen-15-9-s005.docx]

**SUPPLEMENTARY MATERIAL** - **Appendix A5**

Contingency Plan and Risk Analysis

1. **Participant Recruitment**

a) **Risk of Selection Bias**: The sample may not adequately represent the target population, potentially affecting generalizability.
Mitigation Strategy: Define clear, objective inclusion and exclusion criteria. Employ diversified recruitment channels to reach a broad and representative pool of eligible participants.

b) **Risk of Recruitment Difficulties**: Failure to recruit the necessary number of participants within the projected timeline could jeopardize study validity and completion.

Mitigation Strategy: Conduct a prior feasibility assessment and implement proactive recruitment strategies (e.g., institutional campaigns targeting the student population).

1. **Logistical Challenges in Sample Collection**

**Risk**: Delays or non-compliance due to scheduling conflicts, transport limitations, long waiting times, or participant discomfort.

Mitigation Strategy: Offer flexible scheduling, provide transportation support if needed, reduce waiting times, and ensure participant-centred care by trained staff.

1. **Biological Sample Integrity (Blood and Stool)**

**Risk**: Contamination or degradation during collection, transport, or storage, which could compromise data quality.

Mitigation Strategy: Use standardized, sterile collection protocols; ensure cold-chain logistics and validated storage conditions (e.g., −80 °C for stool samples).

1. **Participant Withdrawal**

**Risk**: Attrition during the study may reduce statistical power and lead to incomplete data.

Mitigation Strategy: Strengthen participant engagement through regular communication, appropriate incentives (e.g., gift vouchers), and fostering a positive, respectful research environment.

1. **Intervention Discontinuation (Prebiotic/Placebo)**

**Risk**: Non-adherence due to mild adverse effects, forgetfulness, or lack of motivation during the 6-week intervention phase.

Mitigation Strategy: Monitor participants for side effects and provide clinical support if necessary. Maintain regular check-ins, emphasize adherence, offer simple and convenient dosing formats (e.g., capsules or powders), and use reminder tools (e.g., digital alerts).
