# Supplementary material for: Assessing the impact of binge drinking and a prebiotic intervention on the gut–brain axis in young adults: protocol for a randomised controlled trial
Source: BMJ Open. 2025 Sep 4;15(9):e095932. doi: 10.1136/bmjopen-2024-095932 (PMC12414231; doi:10.1136/bmjopen-2024-095932)
Supplement: Supplementary file 6 [file bmjopen-2024-095932.reviewer_comments.pdf]

PEER REVIEW HISTORY

BMJ Open publishes all reviews undertaken for accepted manuscripts. Reviewers are asked to complete a checklist review form and are provided with free text boxes to elaborate on their assessment. These free text comments are reproduced below.

ARTICLE DETAILS

Title (Provisional)

Assessing the impact of binge drinking and a prebiotic intervention on the gut–brain axis in young adults: protocol for a randomised controlled trial

Authors

Prata-Martins, Diogo; Nobre, Clarisse; Almeida-Antunes, Natália; Azevedo, Pedro; Sousa, Sónia S.; Crego, Alberto; Cryan, John; Sampaio, Adriana; Carbia, Carina; López-Caneda, Eduardo

---

VERSION 1 - REVIEW

**Reviewer**	**1**
**Name**	**Leon, Alberto del Cerro**
**Affiliation**	**Complutense University of Madrid, Experimental Psichology**
**Date**	**04-Feb-2025**
**COI**	**None**

The present protocol aims to describe the associations between alcohol consumption, microbiota and performance in 82 young university students based on recent findings that attribute to the gut-brain axis an important role in behavioral regulation. To achieve this objective, they propose to 1) replicate the associations found between BD consumption and alterations in the microbiota, 2) build a model that explains, through dysbiosis in the microbiota, how alcohol influences the immune and cognitive response, as well as the structure and function of the brain, and 3) analyze the possible effects of an intervention with prebiotics in binge drinkers.

Among the virtues of this work we find that it is a novel proposal that contrasts results previously obtained and tries to offer an explanatory model and a proposal for intervention, which are of great interest for basic and applied research. However, certain aspects of the protocol should be clarified:

1. It is mentioned that during the intervention the participants' diet was followed. However, dietary habits are an essential variable in the understanding of microbiota that should be considered from the recruitment phase. In fact, some dietary requirements or preferences

could be a major distinguishing factor among the participants that should be monitored. Were such circumstances taken into account during recruitment, or how will this information be used in future analyses?

2. As mentioned in the text, it is important to consider some demographic variables such as sex in the study of brain structure and function. However, throughout the methodology described for the realization of the explanatory model with structural equations, there is no mention of the use of demographic variables that could have an important weight in consumption and eating behaviors.

3. In the description of the theoretical model underlying the structural equations, the role played by some of the latent variables introduced, such as alcohol use patterns and immune response is omitted.

Finally, I will make some suggestions that could improve the comprehension and clarity of the text:

1. A follow-up of cognitive tests is mentioned in the description of the methods. However, this follow-up is not reflected in Figure 1.

2. The acronym GM is used for gut microbiota shortening, but in neuroimaging it is usually used for gray matter. Since it is only used once, I suggest dispensing with these acronyms.

3. The methodology used in the neuroimaging analysis would be more convenient to place it in the main text rather than in supplementary material. If it is too long, it could be described in general terms and extended later in the supplementary material.

**Reviewer**	**2**
**Name**	**Asatryan, L**
**Affiliation**	**University of Southern California**
**Date**	**07-Apr-2025**
**COI**	**None**

The study premise is well justified and addresses an important issue of binge drinking in youth and a mitigation approach by potentially targeting gut microbiome dysfunction; this is nicely within the nicely written introduction. All the other required parts for a study protocol are in place, including Materials and Methods, Assessment protocol and Statistical analysis. These parts detail all the necessary information on the study design, study subjects, end point assessments and approaches to statistical analyses. More details pertaining the study procedures are also presented in several Appendices presented at the end of the manuscript. Importantly, authors address potential shortcomings of the study approaches

and have plans ways to address/mitigate (Appendices). A few areas to be addressed before publication.

1. Authors posed questions after the goals (Aims, Hypothesis) which seems to be redundant with the objectives and can be integrated within those.

2. “Alcohol-induced cognitive/brain anomalies” – not clear what authors refer to. If it means anatomical or functional changes, then it has to state so. "anomaly" can be revised.

3. No indication whether data from males and females be pooled or will be analyzed separately. If separately, then what would be the statistical justification for the sample size and whether the proposed sample size is statistically sufficient to analyze data for each gender.

4. The BD group is planned to be identified based on survey assessments. How would these assessments be verified. Will alcohol exposure be determined using tests such as phosphatidyl ethanol?

5. How is the compliance of taking the intervention (doses) being assured? Will a stool test be utilized to detect the presence of inulin and used to verify intervention compliance?

6. The paper needs to be proofread to correct a few typos and grammar errors.

VERSION 1 - AUTHOR RESPONSE

Reviewers' Comments

Reviewer 1 - Dr. Alberto del Cerro Leon, Complutense University of Madrid

The present protocol aims to describe the associations between alcohol consumption, microbiota and performance in 82 young university students based on recent findings that attribute to the gut-brain axis an important role in behavioral regulation. To achieve this objective, they propose to:

1) replicate the associations found between BD consumption and alterations in the microbiota;

2) build a model that explains, through dysbiosis in the microbiota, how alcohol influences the immune and cognitive response, as well as the structure and function of the brain;

3) analyse the possible effects of an intervention with prebiotics in binge drinkers. Among the virtues of this work we find that it is a novel proposal that contrasts results previously obtained and tries to offer an explanatory model and a proposal for intervention, which are of great interest for basic and applied research.

However, certain aspects of the protocol should be clarified:

1. It is mentioned that during the intervention the participants' diet was followed. However,

dietary habits are an essential variable in the understanding of microbiota that should be considered from the recruitment phase. In fact, some dietary requirements or preferences could be a major distinguishing factor among the participants that should be monitored. Were such circumstances taken into account during recruitment, or how will this information be used in future analyses?

R: We appreciate the reviewer's insightful comment regarding the importance of dietary habits in understanding the gut microbiota. We would like to clarify that this variable is indeed taken into account from the recruitment phase of the study. Specifically, one of the exclusion criteria applied during recruitment involves assessing adherence to specific dietary practices, such as a vegan diet. Participants who reported following such a diet are excluded from the study, given its potential to significantly influence gut microbiota composition.

Furthermore, during the clinical interview, a food frequency questionnaire is administered to characterize each participant's habitual dietary pattern (1). This information will facilitate a more accurate interpretation of microbiota composition data, considering individual differences in dietary intake.

Finally, throughout the intervention phase, participants' dietary habits are monitored weekly through the completion of a food diary (1,2). In this diary, all foods and beverages consumed over three days —two weekdays and one weekend day- are recorded. This approach aims to ensure rigorous control of dietary variables, allowing for their appropriate consideration in subsequent analyses.

These methodological details have now been explicitly included in the revised version of the manuscript to clarify how dietary habits are accounted for throughout the study (section *2.4.2.1 Stool samples*, lines 314-319). Individually, the food frequency questionnaire is described in section *2.1.2. Clinical Interview*, lines 201-203, and the food diary in section *2.1.4. Intervention*, lines 228-230.

References

1. Lopes C, Aro A, Azevedo A, Ramos E, Barros H. Intake and adipose tissue composition of fatty acids and risk of myocardial infarction in a male Portuguese community sample. J Am Diet Assoc [Internet]. 2007 Feb [cited 2024 Jul 29];107(2):276–86. Available from: https://pubmed.ncbi.nlm.nih.gov/17258965/

2. Lopes C. Alimentação e Enfarte Agudo do Miocárdio: Estudo caso-controlo de base comunitária. Dissertação de doutoramento apresentada à Faculdade de Medicina da Universidade do Porto. [Internet]. 2000. Available from: https://hdl.handle.net/10216/9938

2. As mentioned in the text, it is important to consider some demographic variables such as sex in the study of brain structure and function. However, throughout the methodology described for the realization of the explanatory model with structural equations, there is no mention of the use of demographic variables that could have an important weight in consumption and eating behaviors.

R: We thank the reviewer for this pertinent and constructive comment. We agree that demographic variables such as sex and age may influence alcohol use, dietary habits, and brain-related outcomes, and should therefore be properly accounted for. In the present study, participants in the control and intervention groups will be matched for sex and age to minimize potential confounding effects at the recruitment level.

Additionally, as clarified in the revised version of the manuscript (Section 2.5. *Statistical Analysis Plan*), these variables will be statistically controlled in all relevant analyses. Specifically, sex and age will be included as covariates across models assessing microbiota, immune markers, cognitive performance, and brain structure and function. This includes the Structural Equation Modeling (SEM), where they will be entered as covariates to adjust for their potential influence on the relationships among latent variables.

These clarifications have been explicitly incorporated into the revised manuscript (Section 2.5. *Statistical Analysis Plan*), both through the addition of a general statement at the beginning of the section regarding the inclusion of sex and age as covariates across all analyses (lines 466-472), and through a specific reference within the description of the SEM model indicating that these variables will also be statistically controlled (lines 482-501).

3. In the description of the theoretical model underlying the structural equations, the role played by some of the latent variables introduced, such as alcohol use patterns and immune response is omitted.

R: We thank the reviewer for this insightful comment, which helped us clarify the theoretical assumptions underlying the proposed structural equation model. In the revised version of the manuscript, we have now elaborated on the expected role of each latent variable within the model. Specifically, *Alcohol Use Patterns* are conceptualized as an exogenous latent variable, reflecting individual differences in drinking behavior that are hypothesized to influence gut microbiota composition and inflammatory processes. *Gut Microbiota* and *Immune Response* are modelled as mediators through which alcohol-related effects may impact brain structure, brain function, and cognitive performance. This formulation is grounded in previous findings highlighting the role of chronic alcohol use in inducing gut dysbiosis and systemic inflammation (1), which may contribute to neuroinflammatory processes and cognitive impairment (2,3). This clarification has now been explicitly incorporated into the revised manuscript (Section *2.5. Statistical Analysis Plan*, lines 490-501).

<u>References</u>

1. Leclercq S, De Timary P, Delzenne NM, Stärkel P. The link between inflammation, bugs, the intestine and the brain in alcohol dependence. Transl Psychiatry 2017 72 [Internet]. 2017 Feb 28 [cited 2023 Mar 30];7(2):e1048–e1048. Available from: https://www.nature.com/articles/tp201715

2. Carbia C, Lannoy S, Maurage P, López-Caneda E, O'Riordan KJ, Dinan TG, et al. A biological framework for emotional dysregulation in alcohol misuse: from gut to brain. Mol Psychiatry 2020 264 [Internet]. 2021 Dec 7 [cited 2023 Mar 30];26(4):1098–118. Available from: https://www.nature.com/articles/s41380-020-

00970-6

3. Carbia C, Bastiaanssen TFS, Iannone LF, García-Cabrerizo R, Boscaini S, Berding K, et al. The Microbiome-Gut-Brain axis regulates social cognition & craving in young binge drinkers. eBioMedicine [Internet]. 2023 Mar 1 [cited 2023 Mar 23];89:104442. Available from: https://www.thelancet.com/journals/ebiom/article/PIIS2352-3964(23)00007-5/abstract

Reviewer: 2 - Dr. L Asatryan, University of Southern California

1. Authors posed questions after the goals (Aims, Hypothesis) which seems to be redundant with the objectives and can be integrated within those.

R: We appreciate the reviewer's observation. We agree that the research questions, as previously presented, closely mirrored the stated objectives and hypotheses, resulting in some redundancy. Accordingly, we have removed the standalone questions and integrated their content directly into the hypotheses, which now encapsulate both the research aims and the key questions guiding the study. This adjustment improves the conciseness and clarity of the section. The change has been implemented in the revised version of the manuscript (Section *1.1. Aims and Hypotheses*, lines 141-171).

2. "Alcohol-induced cognitive/brain anomalies" – not clear what authors refer to. If it means anatomical or functional changes, then it has to state so. "anomaly" can be revised.

R: We thank the reviewer for the observation regarding the ambiguity of the expression "alcohol-induced cognitive/brain anomalies." We agree that the term "anomalies" may lead to imprecise interpretations, and therefore, the expression has been revised and replaced with "alcohol-related cognitive and brain impairments" (Section *1.1. Aims and Hypotheses*, lines 149-150). We believe this revised formulation more clearly and accurately conveys that we are referring to alcohol-related changes that affect both brain structure and function, in line with the terminology commonly used in the scientific literature in this field.

3. No indication whether data from males and females be pooled or will be analyzed separately. If separately, then what would be the statistical justification for the sample size and whether the proposed sample size is statistically sufficient to analyze data for each gender.

R: We thank the reviewer for raising this important point. In the present study, male and female participants will be matched across groups in terms of age and sex to minimize demographic confounding. In the main analyses, data from both sexes will be analyzed jointly, with sex included either as a covariate or as a between-subjects factor, depending on the

statistical model (as specified in the revised version of the manuscript, in *2.5. Statistical Analysis Plan*).

Should significant interactions with sex emerge in any of the outcome domains, we will conduct follow-up exploratory analyses stratified by sex. However, we acknowledge that the study was not powered to detect sex-specific effects, and any subgroup analyses will be interpreted with caution. This approach allows us to account for sex-related variance while maintaining statistical power in the primary models.

In response to this comment —and in alignment with the related observation from Reviewer 1- we have now explicitly indicated in the Statistical Analysis section of the revised manuscript that sociodemographic variables, such as sex and age, will be included in the statistical models across all outcome domains, including the structural equation model.

4. The BD group is planned to be identified based on survey assessments. How would these assessments be verified. Will alcohol exposure be determined using tests such as phosphatidyl ethanol?

R: We thank the reviewer for the opportunity to address this point. Participants in the binge drinking group are identified during the initial recruitment phase based on their responses to the Alcohol Use Disorders Identification Test (AUDIT). To ensure consistency and reliability, the AUDIT is administered again during the clinical interview phase, along with additional questions regarding alcohol intake during the previous week, including the quantity and type of beverages consumed. This procedure provides both a standardized assessment and a detailed estimate of recent alcohol use. We note that no biochemical tests, such as phosphatidylethanol (PEth), are planned to verify alcohol exposure, as the identification of BD status in this study is based on validated self-report measures consistent with previous research protocols in this population (1,2).

<u>References</u>

1. Almeida-Antunes N, Vasconcelos M, Crego A, Rodrigues R, Sampaio A, López-Caneda E. Forgetting Alcohol: A Double-Blind, Randomized Controlled Trial Investigating Memory Inhibition Training in Young Binge Drinkers. Front Neurosci. 2022 Jun 29;16:914213.
2. Lees B, Mewton L, Stapinski L, Squeglia LM, Rae C, Teesson M. Binge drinking in young people: Protocol for a systematic review of neuropsychological, neurophysiological and neuroimaging studies. BMJ Open [Internet]. 2018 Jul 1 [cited 2025 May 15];8(7). Available from: https://www.pubmed.ncbi.nlm.nih.gov/30061448/

5. How is the compliance of taking the intervention (doses) being assured? Will a stool test be utilized to detect the presence of inulin and used to verify intervention compliance?

R: We thank the reviewer for the comment and would like to clarify the procedures adopted to promote adherence to the intervention. We acknowledge that no objective or direct

method has been established to confirm strict compliance with the daily intake of the intervention. As is common in nutritional and behavioral studies, we rely on the participants' motivation and commitment to follow the recommended protocol.

To facilitate adherence and minimize forgetfulness, each participant receives individual containers clearly labelled with the specific date for each dose. These containers include the exact daily dose of inulin or maltodextrin, allowing for structured and easy-to-follow intake. In addition, participants receive the doses in two-week batches and attend biweekly meetings with the research team to provide feedback and collect the next set of containers. This regular contact helps monitor engagement and reinforces adherence throughout the study period.

In response to the reviewer's concern, a paragraph summarizing these procedures has now been added to Section 2.1.4. *Intervention* (lines 227-235) of the revised manuscript.

6. The paper needs to be proofread to correct a few typos and grammar errors.

R: We thank the reviewer for their careful and attentive reading of the manuscript. In response, the text has been thoroughly proofread to correct typographical and grammatical errors. Minor issues such as the incorrect use of "broadcasted" (now corrected to "broadcast"), a typographical error in "stoll samples" (now "stool samples"), and occasional agreement errors (e.g., "the effects... is" revised to "the effects... are") have been addressed to improve overall clarity and accuracy.

VERSION 2 - REVIEW

**Reviewer**	**1**
**Name**	**Leon, Alberto del Cerro**
**Affiliation**	**Complutense University of Madrid, Experimental Psichology**
**Date**	**09-Jul-2025**
**COI**	

The comments raised during the review process have been satisfactorily addressed. I would, however, recommend including the exclusion criteria related to restricted dietary habits within the manuscript for greater clarity. Overall, the manuscript now demonstrates excellent quality and is fully deserving of publication.

**Reviewer**	**2**
**Name**	**Asatryan, L**
**Affiliation**	**University of Southern California**

Date **08-Jul-2025**

COI

Thank you for sufficiently addressing the review concerns.

VERSION 2 - AUTHOR RESPONSE

Reviewers' Comments

Reviewer 1

1.The comments raised during the review process have been satisfactorily addressed. I would, however, recommend including the exclusion criteria related to restricted dietary habits within the manuscript for greater clarity. Overall, the manuscript now demonstrates excellent quality and is fully deserving of publication.

R: We would like to thank the reviewer for the positive evaluation of the manuscript and for the valuable suggestion regarding the need to clarify the exclusion criteria related to dietary habits.

In response to this recommendation, the exclusion criteria have been rewritten to ensure greater clarity and comprehensiveness, with special attention given to dietary habits and medicine consumption. The changes are reflected in section *2.2. Study Population* of the manuscript, lines 261 to 279.

Reviewer: 2

1.Thank you for sufficiently addressing the review concerns.

R: We sincerely thank the reviewer for their thoughtful and constructive feedback. We truly believe that the manuscript has significantly improved as a result of their comments and suggestions.
